# Changes in Global and Nodal Networks in Patients With Unipolar Depression After 3-Week Repeated Transcranial Magnetic Stimulation Treatment

**DOI:** 10.3389/fpsyt.2019.00686

**Published:** 2019-10-09

**Authors:** Kuk-In Jang, Miseon Shim, Sangmin Lee, Han-Jeong Hwang, Jeong-Ho Chae

**Affiliations:** ^1^Department of Psychiatry, College of Medicine, The Catholic University of Korea, Seoul, South Korea; ^2^Emotion Laboratory, Department of Psychiatry, College of Medicine, The Catholic University of Korea, Seoul, South Korea; ^3^Department of Psychiatry, University of Missouri-Kansas City, Center for Behavioral Medicine, Kansas, MO, United States; ^4^Department of Medical IT Convergence Engineering, Kumoh National Institute of Technology, Gumi, South Korea

**Keywords:** unipolar depression, repeated transcranial magnetic stimulation, electroencephalogram, cortical source network, brain stimulation

## Abstract

**Objectives:** Repeated transcranial magnetic stimulation (rTMS) therapy has been applied in depressive disorders, but its neurobiological effect has not been well understood. Changes in cortical source network after treatment need to be confirmed. The present study investigated the effect of 3-week rTMS therapy on the symptom severity and cortical source network in patients with unipolar depression.

**Methods:** Thirty-five patients with unipolar major depressive disorder participated in the study. High-frequency (10 Hz) rTMS was applied at the left dorsolateral prefrontal cortex during 3 weeks (five consecutive weekdays every week). Clinical symptoms were examined using the Hamilton Rating Scale for Depression and Anxiety. The resting state electroencephalography was recorded with 62 scalp channels before and after rTMS treatment.

**Results:** Clinical symptoms significantly improved after rTMS treatment in both the active (p = 0.001) and sham groups (p = 0.002). However, an increased cortical source network in global and nodal levels was observed only in the active group after a 3-week treatment.

**Conclusions:** The present study indicates that rTMS treatment leads to improved symptoms in patients with unipolar depression. Furthermore, treatment outcome of real effect was assured in changes of cortical source network.

## Introduction

Repeated transcranial magnetic stimulation (rTMS) has been proposed as an alternative treatment for depression (1, 2), and it has also been applied in other neuropsychiatric disorders (3–5). Basically, rTMS affects the neuronal polarization in the cytoplasmic membrane, which has significant impacts on the brain functions (6). Long-lasting effects on depression have been observed after applying high-frequency (10 to 20 Hz) rTMS at the dorsolateral prefrontal cortex (DLPFC) with multiple sessions in 10 to 15 consecutive days (7). Additionally, the primary effects of rTMS therapy have been established in medication-resistant patients with major depressive disorder (MDD) (8). Therefore, rTMS may have a significant positive anti-depressive effect in patients with depression (9).

One previous study has demonstrated that rTMS therapy increases the connectivity of default mode regions, such as subgenual anterior cingulate cortex (sgACC), in depressive patients with traumatic brain injury (10). High-frequency stimulation applied over the left prefrontal cortex induces an enhanced theta–gamma coupling (11) and modulates the resting state functional connectivity between the DLPFC and the limbic lobe (12). In addition, accelerated high-frequency rTMS improves functional connectivity in the sgACC region in patients with treatment-resistant unipolar depression (13). Regional volume reduction of the sgACC has been demonstrated in depressive patients compared with bipolar disorder patients or healthy controls (14), and abnormal network homogeneity of default mode regions, such as precuneus and posterior cingulate cortex (PCC), has also been observed in patients with depression (15). However, the treatment effect of rTMS in patients with unipolar depression remains unclear how the clinically configured magnetic stimulation affects the cortical source region.

Electroencephalogram (EEG) has been considered a reliable approach to analyze the cortical network (16). The antidepressant effect is indicated of changes in neural network. (17). Furthermore, there is no study exploring changes of cortical source level network in patients with depression with 3-week treatment of rTMS. In this study, we investigated the effect of rTMS treatment on the cortical source network in patients with unipolar depression using EEG based on the graph theory. We hypothesized that patients with MDD would show significant changes in network measurements and symptom severity after rTMS treatment. The findings would help understand the neurobiological mechanisms underlying the effects of rTMS.

## Materials and Methods

### Participants

Thirty-five patients with unipolar MDD participated in the study, and they were randomly classified into the active (19 participants: 4 men and 15 women) and sham (16 participants: 5 men and 11 women) groups. The age of all participants ranged between 18 and 65 years, and the mean age of participants was 33.53 ± 12.89 years in the active group and 35.00 ± 11.94 years in the sham group (**Table 1**). Drug information, education level, and frequency of physical activity are also shown in **Table 1**. The present study was conducted between February 2015 and November 2016. All participants were native Korean and were diagnosed with unipolar MDD according to the 4th edition of the *Diagnostic and Statistical Manual of Mental Disorders* (*DSM-4*). There are very few differences in the diagnosis of the patient with depression between *DSM-4* and *DSM-5*. Clinically structured interviews were performed using the Mini-International Neuropsychiatric Interview (18) by a psychiatrist who was blind to the present study design. Ten participants were administered antidepressants during the study period (**Table 1**). The participants with other current and/or lifetime Axis I psychiatric disorders; history of epilepsy, spontaneous seizures, or brain surgery; substance use; or pregnancy were excluded from this study. Participants with contraindications for magnetic stimulation (e.g., cardiac pace makers, implanted medication pumps, or hearing aids consisting of metallic materials) were also excluded from this study. For safety purposes, all participants underwent a brief EEG session to screen for epileptiform EEG abnormalities before rTMS. The Institutional Review Board of Seoul St. Mary’s Hospital, College of Medicine, The Catholic University of Korea approved the study protocol (approval number: KC14DDSE0479). All participants provided written informed consent.

**Table 1:** Demographic data of the participants in the present study.

Variables	ACTIVE rTMS (*n* = 19)	SHAM rTMS (*n* = 16)	*t* or *χ^2^*
	Mean (standard deviation)		
Age	33.53 (12.89)	35.00 (11.94)	*p* = 0.730
Sex (n, male/female)	4/15	5/11	*p* = 0.700
Education	13.26 (1.66)	13.33 (2.69)	*p* = 0.926
Physical activity	2.32 (1.80)	2.13 (1.63)	*p* = 0.746
Medication			
* Amitriptyline*	3		
* Escitalopram*	1		
* Fluoxetine*	1	1	
* Mirtazapine*	1		
* Paroxetine*	1	2	
* Sertraline*	1	1	

Physical activity: 0: not at all, 1: one to two times a month, 2: three to four times a month, 3: one to two times a week, 4: three to four times a week, 5: not less than five times a week.

### Clinical Assessments

#### Hamilton Rating Scales for Depression (HAM-D) and Anxiety (HAM-A)

The HAM-D and HAM-A were rated by a psychiatrist who was blind to the treatment groups. The HAM-D (19) and HAM-A (20) consist of 17 and 14 items, respectively.

#### rTMS Protocol

rTMS was conducted using a device named TAMAS (REMED, Daejeon, Korea) with a figure-of-eight-shaped coil. Before each rTMS session, the motor threshold (MT) was determined by stimulating the motor cortex with the lowest amount of energy required to produce five consecutive twitches of the right abductor pollicis brevis (APB) muscle. The stimulation was applied at 110% of the individual MT. The average stimulation intensity for all participants was 61.91 ± 21.06% of the maximal stimulator output. The stimulation was applied over the DLPFC, and the stimulation location was determined by moving the TMS coil 5 cm anterior to the optimal surface site for activation of the right APB muscle. The frequency of stimulation was set at 10 Hz for 5 s, with an intertrain interval of 25 s. Treatment sessions lasted for 30 min (60 trains) and included 3,000 pulses. Sham stimulation was performed using a sham coil, which elicited no tactile sensation at the site of stimulation and induced no cortical stimulation, and thus provided only matched acoustic sensation. Each participant underwent 15 rTMS sessions on 15 consecutive weekdays.

#### Electrophysiological Measurement and Preprocess

Participants were seated in a comfortable chair in a sound-attenuated room. The resting-state EEG was recorded with eyes closed for 5 min. EEG data were recorded using a NeuroScan SynAmps amplifier (Compumedics USA, El Paso, TX, USA) with a head cap mounted with AgCl electrodes according to an extended international 10-20 system. We recorded EEG data from 62 scalp positions (FP1, FPZ, FP2, AF3, AF4, F7, F5, F3, F1, FZ, F2, F4, F6, F8, FT7, FC5, FC3, FC1, FCZ, FC2, FC4, FC6, FT8, T7, C5, C3, C1, CZ, C2, C4, C6, T8, TP7, CP5, CP3, CP1, CPZ, CP2, CP4, CP6, TP8, P7, P5, P3, P1, PZ, P2, P4, P6, P8, PO7, PO5, PO3, POZ, PO4, PO6, PO8, CB1, O1, OZ, O2, and CB2). Additional electrodes were placed above and below the left eye for vertical electrooculogram recording and at the outer canthus of each eye for horizontal electrooculogram recording. EEG data were recorded with a 1- to 100-Hz bandpass filter at a sampling rate of 1,000 Hz. The signals were referenced to both mastoids where the ground electrode was placed on the forehead. Impedance between the electrodes and scalp was maintained below 5 kΩ during the entire recording session. EEG data were preprocessed using Scan 4.5 software and Curry suite 7.0 (Compumedics USA, El Paso, TX, USA). The EEG data were bandpass filtered at 0.1 to 60 Hz. Gross artifacts, such as eye-related and muscle artifacts, were corrected using independent component analysis implemented with a multiple artifact rejection algorithm (21). After the removal of artifacts, the data were segmented into epochs with a duration of 10 s, and the epoch was rejected if it contained significant physiological artifacts (amplitude > 100 μV) at any sites over all electrodes. A total of 12 artifact-free epochs (2 min) were used for each subject for the source-level network analysis. It was demonstrated that the length of epoched EEG data (2 min) is sufficient for functional connectivity (22).

##### Source Localization

The source model, constructed from the Colin 27 standard template brain, consisted of 15,000 cortical vertices in both hemispheres. The three-layer (inner skull, outer skull, and the scalp) boundary element method (BEM) model for creating a lead field matrix was generated using the Open MEEG implemented in Brainstorm (https://neuroimage.usc.edu/brainstorm/) (23). A time series of source activity at the cortical vertex was evaluated using the weighted minimum-norm estimation method. After computing time series at each vertex, the representative signals of 68 region of interests (ROIs) based on the Desikan–Killiany Atlas (24) were estimated by principal component analysis. A time series of the cortical sources at each of the 68 ROIs was bandpass filtered (1–55 Hz) and divided into seven frequency bands [delta (1–4 Hz), theta (4–8 Hz), alpha (8–12 Hz), low beta (12–18 Hz), mid beta (18–22 Hz), high beta (22–30 Hz), and gamma (30–55 Hz)].

### Connectivity and Network Analysis

Phase locking value (PLV) based on the Hilbert transform was computed to evaluate functional connectivity between each pair of nodes in the whole brain (25). The value of PLV is related to the strength of the functional connection between two nodes. If PLV is approached to between two nodes, the strength of the functional connection is stronger than other pairs of nodes. A raw PLV matrix was used as an adjacency matrix for weighted network analysis.

Moreover, we computed various weighted network measures based on graph theory (26). We investigated the brain network using two different perspectives, i.e., “global level” and “nodal level.” The global-level values represent the characteristics of a whole-brain network while the nodal-level values indicate the properties at each node (specific brain regions). We assessed a total of four different types of global-level network measures as follows: 1) strength, 2) clustering coefficient, 3) path length, and 4) efficiency. Additionally, the clustering coefficient and the efficiency at each node were evaluated for nodal-level analysis. All network measures were computed using the Brain Connectivity Toolbox (BCT, http://www.brain-connectivity-toolbox.net), an open Matlab source.

### Statistical Analyses

Descriptive statistics were performed between the active and control groups using *t* test and chi-square test. A p value of less than 0.05 was considered statistically significant for two-tailed tests. The effects of rTMS treatment in each group were compared using the paired *t* tests. In the comparison of nodal levels, the *p* value was adjusted using the false discovery rate correction; the effect size was calculated using Cohen’s *d*, and a Cohen’s *d* value of >0.60 was considered significant.

## Results

**Figure 1:**
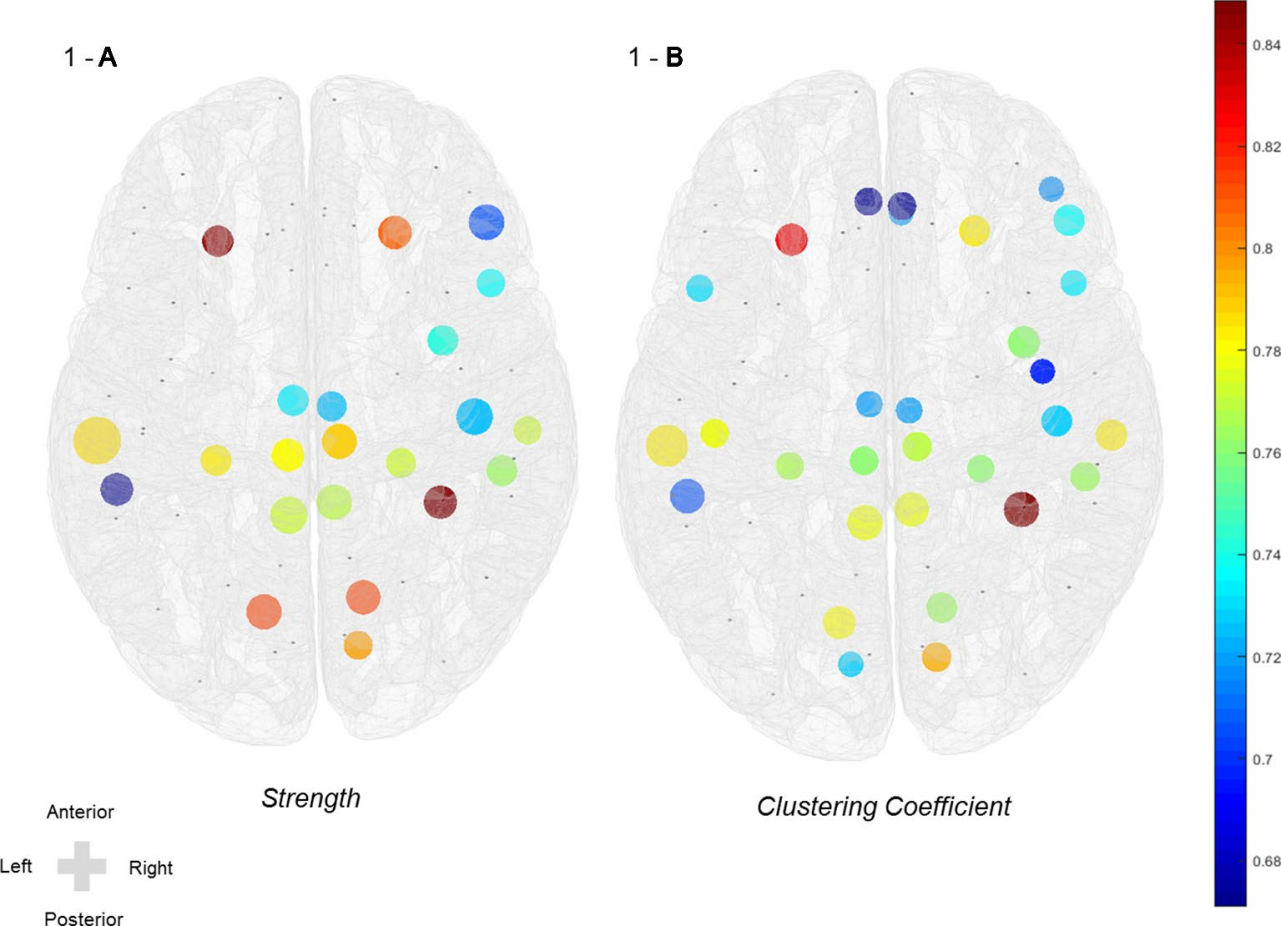
Increased nodal strength and clustering coefficient of mid-beta frequency in the active group after rTMS treatment. **(A)** Nodal strength and **(B)** nodal clustering coefficient in mid-beta frequency. Color scale indicates effect size (Cohen’s *d* value > 0.60).

Symptomatic differences were found between baseline and 3 weeks after rTMS treatment in both active and sham rTMS groups. In the active group, Hamilton depression and anxiety scores significantly decreased 3 weeks after treatment compared with the baseline (HAM-D: 21.00 ± 5.12 vs 15.47 ± 6.32, *p* = 0.001; HAM-A: 23.47 ± 7.38 vs 16.79 ± 6.88, *p* = 0.001; **Table 2**). In the sham group, Hamilton depression and anxiety scores also significantly decreased after 3 weeks compared with baseline (HAM-D: 19.31 ± 6.10 vs 15.38 ± 6.18, *p* = 0.002; HAM-A: 21.75 ± 8.01 vs 16.13 ± 7.33, *p* < 0.001; **Table 2**). Network analyses revealed significant differences between baseline and post-treatment in the active group. The global efficiency in delta frequency significantly increased after rTMS treatment (0.55 ± 0.06 vs 0.58 ± 0.07, *p* = 0.044). The global strength, clustering coefficient, and efficiency in theta frequency increased after rTMS treatment (strength: 34.58 ± 5.59 vs 37.87 ± 6.16, *p* = 0.034; clustering coefficient: 0.47 ± 0.10 vs 0.52 ± 0.11, *p* = 0.043; efficiency: 0.55 ± 0.07 vs 0.59 ± 0.08, *p* = 0.025; **Table 2**). The global strength, clustering coefficient, and efficiency in low-beta frequency increased after treatment (strength: 34.67 ± 5.98 vs 38.21 ± 6.27, *p* = 0.025; clustering coefficient: 0.46 ± 0.10 vs 0.52 ± 0.11, *p* = 0.038; efficiency: 0.55 ± 0.07 vs 0.59 ± 0.07, *p* = 0.021; **Table 2**). The global strength, clustering coefficient, and efficiency in mid-beta frequency increased after rTMS treatment (strength: 36.36 ± 6.15 vs 40.28 ± 5.75, *p* = 0.033; clustering coefficient: 0.49 ± 0.10 vs 0.56 ± 0.10, *p* = 0.033; efficiency: 0.57 ± 0.07 vs 0.62 ± 0.07, *p* = 0.037; **Table 2**). Nodal strength and clustering coefficient in mid-beta frequency significantly increased after treatment. In the nodal strength analysis, the right fusiform (38.91 ± 8.34 vs 45.10 ± 5.94, *p* = 0.049, Cohen’s *d* = 0.85), left inferior temporal (38.59 ± 8.90 vs 44.18 ± 7.82, *p* = 0.049, Cohen’s *d* = 0.67), right inferior temporal (41.13 ± 7.55 vs 45.97 ± 4.77, *p* = 0.049, Cohen’s *d* = 0.77), right insula (39.84 ± 7.39 vs 44.94 ± 4.90, *p* = 0.049, Cohen’s *d* = 0.74), left isthmus cingulate (40.03 ± 7.54 vs 44.98 ± 4.99, *p* = 0.049, Cohen’s *d* = 0.77), right isthmus cingulate (39.99 ± 7.49 vs 44.89 ± 4.94, *p* = 0.049, Cohen’s *d* = 0.77), left lateral orbitofrontal (40.15 ± 8.62 vs 46.13 ± 4.96, *p* = 0.049, Cohen’s *d* = 0.85), right lateral orbitofrontal (40.93 ± 7.03 vs 45.98 ± 5.26, *p* = 0.049, Cohen’s *d* = 0.81), left lingual (38.52 ± 7.28 vs 44.42 ± 7.15, *p* = 0.049, Cohen’s *d* = 0.82), right lingual (38.86 ± 6.79 vs 44.45 ± 6.90, *p* = 0.049, Cohen’s *d* = 0.82), left middle temporal (39.52 ± 8.31 vs 45.62 ± 7.10, *p* = 0.049, Cohen’s *d* = 0.79), right middle temporal (40.68 ± 7.82 vs 45.64 ± 4.55, *p* = 0.049, Cohen’s *d* = 0.77), left paracentral (42.04 ± 7.07 vs 46.78 ± 4.86, *p* = 0.049, Cohen’s *d* = 0.78), right paracentral (42.17 ± 7.21 vs 47.07 ± 4.95, *p* = 0.049, Cohen’s *d* = 0.79), left parahippocampal (42.14 ± 6.99 vs 46.87 ± 4.85, *p* = 0.049, Cohen’s *d* = 0.79), right parahippocampal (42.07 ± 6.78 vs 46.65 ± 4.88, *p* = 0.049, Cohen’s *d* = 0.77), right parsopercularis (41.98 ± 6.98 vs 46.50 ± 5.15, *p* = 0.049, Cohen’s *d* = 0.74), right parstriangularis (40.88 ± 8.34 vs 45.98 ± 5.96, *p* = 0.049, Cohen’s *d* = 0.70), right pericalcarine (40.86 ± 6.50 vs 45.68 ± 5.47, *p* = 0.049, Cohen’s *d* = 0.80), right postcentral (41.59 ± 8.12 vs 46.86 ± 6.27, *p* = 0.049, Cohen’s *d* = 0.73), left posterior cingulate (43.09 ± 6.95 vs 47.75 ± 5.70, *p* = 0.049, Cohen’s *d* = 0.73), and right posterior cingulate (43.10 ± 6.95 vs 47.73 ± 5.77, *p* = 0.049, Cohen’s *d* = 0.72) regions significantly increased after rTMS treatment (**Figure 1** and **Table 3**). In nodal clustering coefficient analysis, the right fusiform (0.51 ± 0.12 vs 0.61 ± 0.09, *p* = 0.049, Cohen’s *d* = 0.85), left inferior temporal (0.52 ± 0.12 vs 0.60 ± 0.11, *p* = 0.049, Cohen’s *d* = 0.71), right inferior temporal (0.53 ± 0.12 vs 0.61 ± 0.08, *p* = 0.049, Cohen’s *d* = 0.76), right insula (0.52 ± 0.12 vs 0.60 ± 0.08, *p* = 0.049, Cohen’s *d* = 0.76), left isthmus cingulate (0.52 ± 0.12 vs 0.61 ± 0.08, *p* = 0.049, Cohen’s *d* = 0.78), right isthmus cingulate (0.52 ± 0.12 vs 0.60 ± 0.08, *p* = 0.049, Cohen’s *d* = 0.78), left lateral orbitofrontal (0.53 ± 0.13 vs 0.62 ± 0.09, *p* = 0.049, Cohen’s *d* = 0.83), right lateral orbitofrontal (0.53 ± 0.12 vs 0.62 ± 0.09, *p* = 0.049, Cohen’s *d* = 0.78), left lingual (0.51 ± 0.11 vs 0.60 ± 0.11, *p* = 0.049, Cohen’s *d* = 0.78), right lingual (0.52 ± 0.10 vs 0.60 ± 0.11, *p* = 0.049, Cohen’s *d* = 0.76), right medial orbitofrontal (0.53 ± 0.12 vs 0.61 ± 0.10, *p* = 0.049, Cohen’s *d* = 0.72), left middle temporal (0.52 ± 0.12 vs 0.61 ± 0.10, *p* = 0.049, Cohen’s *d* = 0.78), right middle temporal (0.53 ± 0.12 vs 0.61 ± 0.08, *p* = 0.049, Cohen’s *d* = 0.78), left paracentral (0.54 ± 0.11 vs 0.62 ± 0.09, *p* = 0.049, Cohen’s *d* = 0.76), right paracentral (0.54 ± 0.12 vs 0.62 ± 0.09, *p* = 0.049, Cohen’s *d* = 0.77), left parahippocampal (0.54 ± 0.11 vs 0.62 ± 0.09, *p* = 0.049, Cohen’s *d* = 0.77), right parahippocampal (0.54 ± 0.11 vs 0.62 ± 0.09, *p* = 0.049, Cohen’s *d* = 0.76), left parsopercularis (0.53 ± 0.13 vs 0.62 ± 0.09, *p* = 0.049, Cohen’s *d* = 0.73), right parsopercularis (0.54 ± 0.11 vs 0.62 ± 0.09, *p* = 0.049, Cohen’s *d* = 0.73), right parsorbitalis (0.54 ± 0.12 vs 0.62 ± 0.09, *p* = 0.049, Cohen’s *d* = 0.72), right parstriangularis (0.53 ± 0.12 vs 0.61 ± 0.09, *p* = 0.049, Cohen’s *d* = 0.74), left pericalcarine (0.54 ± 0.12 vs 0.62 ± 0.09, *p* = 0.049, Cohen’s *d* = 0.73), right pericalcarine (0.53 ± 0.11 vs 0.62 ± 0.09, *p* = 0.049, Cohen’s *d* = 0.80), left postcentral (0.53 ± 0.12 vs 0.61 ± 0.10, *p* = 0.049, Cohen’s *d* = 0.78), right postcentral (0.55 ± 0.12 vs 0.63 ± 0.10, *p* = 0.049, Cohen’s *d* = 0.73), left posterior cingulate (0.55 ± 0.11 vs 0.63 ± 0.10, *p* = 0.049, Cohen’s *d* = 0.72), right posterior cingulate (0.55 ± 0.12 vs 0.63 ± 0.10, *p* = 0.049, Cohen’s *d* = 0.72), right precentral (0.53 ± 0.12 vs 0.61 ± 0.11, *p* = 0.049, Cohen’s *d* = 0.70), left rostral anterior cingulate (0.53 ± 0.12 vs 0.61 ± 0.12, *p* = 0.049, Cohen’s *d* = 0.67), and right rostral anterior cingulate (0.53 ± 0.12 vs 0.61 ± 0.12, *p* = 0.049, Cohen’s *d* = 0.67) regions significantly increased after rTMS treatment (**Figure 1** and **Table 3**). 

**Table 2:** Comparison of psychometrics and values of global network between time interval.

Variables	ACTIVE rTMS (*n* = 19)		*p-value*	SHAM rTMS (*n* = 16)		*p-value*
	Baseline	Post		Baseline	**Post**	
	Mean (SD)			Mean (SD)		
Clinical measures						
HAM-D	21.00 (5.12)	15.47 (6.32)	**0.001**	19.31 (6.10)	15.38 (6.18)	**0.002**
HAM-A	23.47 (7.38)	16.79 (6.88)	**0.001**	21.75 (8.01)	16.13 (7.33)	**<0.001**
Delta						
Strength	34.75 (4.60)	37.22 (5.74)	0.065	36.14 (5.24)	34.90 (8.68)	0.615
CC	0.46 (0.07)	0.50 (0.10)	0.106	0.49 (0.08)	0.47 (0.14)	0.693
PL	2.28 (0.29)	2.21 (0.39)	0.346	2.25 (0.30)	2.35 (0.54)	0.48
Efficiency	0.55 (0.06)	0.58 (0.07)	**0.044**	0.56 (0.06)	0.55 (0.11)	0.573
Theta						
Strength	34.58 (5.59)	37.87 (6.16)	**0.034**	35.63 (4.77)	35.20 (7.74)	0.85
CC	0.47 (0.10)	0.52 (0.11)	**0.043**	0.49 (0.08)	0.48 (0.13)	0.875
PL	2.31 (0.37)	2.18 (0.40)	0.131	2.27 (0.28)	2.32 (0.50)	0.723
Efficiency	0.55 (0.07)	0.59 (0.08)	**0.025**	0.57 (0.07)	0.56 (0.10)	0.695
Alpha						
Strength	34.30 (5.96)	6.02 (1.38)	0.063	35.01 (5.87)	34.35 (7.31)	0.772
CC	0.46 (0.10)	0.51 (0.11)	0.081	0.47 (0.09)	0.46 (0.12)	0.891
PL	2.35 (0.42)	2.21 (0.42)	0.213	2.32 (0.32)	2.38 (0.50)	0.701
Efficiency	0.54 (0.08)	0.58 (0.08)	0.051	0.55 (0.07)	0.54 (0.09)	0.677
Low beta						
Strength	34.67 (5.98)	38.21 (6.27)	**0.025**	35.34 (6.37)	35.05 (6.46)	0.891
CC	0.46 (0.10)	0.52 (0.11)	**0.038**	0.47 (0.10)	0.48 (0.10)	0.882
PL	2.31 (0.36)	2.17 (0.42)	0.11	2.29 (0.36)	2.31 (0.43)	0.867
Efficiency	0.55 (0.07)	0.59 (0.07)	**0.021**	0.56 (0.07)	0.55 (0.08)	0.671
Mid beta						
Strength	36.36 (6.15)	40.28 (5.75)	**0.033**	39.55 (6.12)	38.70 (6.52)	0.757
CC	0.49 (0.10)	0.56 (0.10)	**0.033**	0.55 (0.10)	0.53 (0.10)	0.818
PL	2.18 (0.34)	2.01 (0.32)	0.083	2.04 (0.30)	2.11 (0.42)	0.659
Efficiency	0.57 (0.07)	0.62 (0.07)	**0.037**	0.61 (0.08)	0.59 (0.08)	0.685
High beta						
Strength	35.28 (6.88)	38.01 (4.83)	0.086	36.00 (5.06)	35.88 (6.77)	0.946
CC	0.47 (0.11)	0.51 (0.08)	0.113	0.48 (0.08)	0.49 (0.10)	0.811
PL	2.30 (0.43)	2.18 (0.33)	0.188	2.27 (0.26)	2.29 (0.44)	0.832
Efficiency	0.56 (0.08)	0.59 (0.05)	0.085	0.56 (0.06)	0.56 (0.08)	0.722
Gamma						
Strength	33.43 (5.74)	36.05 (5.46)	0.142	33.86 (5.07)	32.59 (7.04)	0.518
CC	0.44 (0.09)	0.48 (0.09)	0.183	0.44 (0.08)	0.43 (0.11)	0.689
PL	2.45 (0.40)	2.34 (0.40)	0.333	2.43 (0.28)	2.53 (0.49)	0.476
Efficiency	0.53 (0.07)	0.57 (0.06)	0.115	0.54 (0.06)	0.52 (0.08)	0.394

Bold, statistical significance at p < 0.05.

**Table 3:** In the active group, increased node values of strength and clustering coefficient in mid-beta frequency after 3 weeks of treatment.

Regions	Strength		Cohen’s *d*	Clustering coefficient		Cohen’s *d*	MNI coordinates		
	Baseline vs post mean (SD)	*p* value	Effect size	Baseline vs postmean (SD)	*p* value	Effect size	*x*	*y*	*z*
Fusiform R	38.91 (8.34) vs 45.10 (5.94)	0.049	0.85	0.51 (0.12) vs 0.61 (0.09)	0.049	0.85	−35.24	−14.92	23.53
Inferior temporal L	38.59 (8.90) vs 44.18 (7.82)	0.049	0.67	0.52 (0.12) vs 0.60 (0.11)	0.049	0.71	52.04	−11.39	24.42
Inferior temporal R	41.13 (7.55) vs 45.97 (4.77)	0.049	0.77	0.53 (0.12) vs 0.61 (0.08)	0.049	0.76	−51.76	−6.39	21.09
Insula R	39.84 (7.39) vs 44.94 (4.90)	0.049	0.74	0.52 (0.12) vs 0.60 (0.08)	0.049	0.76	−35.78	28.61	42.88
Isthmus cingulate L	40.03 (7.54) vs 44.98 (4.99)	0.049	0.77	0.52 (0.12) vs 0.61 (0.08)	0.049	0.78	5.78	−18.50	64.84
Isthmus cingulate R	39.99 (7.49) vs 44.89 (4.94)	0.049	0.77	0.52 (0.12) vs 0.60 (0.08)	0.049	0.78	−6.48	−15.01	65.35
Lateral orbitofrontal L	40.15 (8.62) vs 46.13 (4.96)	0.049	0.85	0.53 (0.13) vs 0.62 (0.09)	0.049	0.83	24.91	55.48	25.30
Lateral orbitofrontal R	40.93 (7.03) vs 45.98 (5.26)	0.049	0.81	0.53 (0.12) vs 0.62 (0.09)	0.049	0.78	−23.06	57.84	24.54
Lingual L	38.52 (7.28) vs 44.42 (7.15)	0.049	0.82	0.51 (0.11) vs 0.60 (0.11)	0.049	0.78	12.42	−44.54	42.23
Lingual R	38.86 (6.79) vs 44.45 (6.90)	0.049	0.82	0.52 (0.10) vs 0.60 (0.11)	0.049	0.76	−14.51	−40.68	43.61
Medial orbitofrontal R	–	–	–	0.53 (0.12) vs 0.61 (0.10)	0.049	0.72	−3.76	62.38	28.58
Middle temporal L	39.52 (8.31) vs 45.62 (7.10)	0.049	0.79	0.52 (0.12) vs 0.61 (0.10)	0.049	0.78	57.52	1.74	31.21
Middle temporal R	40.68 (7.82) vs 45.64 (4.55)	0.049	0.77	0.53 (0.12) vs 0.61 (0.08)	0.049	0.78	−58.64	4.38	30.23
Paracentral L	42.04 (7.07) vs 46.78 (4.86)	0.049	0.78	0.54 (0.11) vs 0.62 (0.09)	0.049	0.76	6.02	−2.21	103.46
Paracentral R	42.17 (7.21) vs 47.07 (4.95)	0.049	0.79	0.54 (0.12) vs 0.62 (0.09)	0.049	0.77	−7.93	1.43	103.05
Parahippocampal L	42.14 (6.99) vs 46.87 (4.85)	0.049	0.79	0.54 (0.11) vs 0.62 (0.09)	0.049	0.77	25.33	−3.60	25.34
Parahippocampal R	42.07 (6.78) vs 46.65 (4.88)	0.049	0.77	0.54 (0.11) vs 0.62 (0.09)	0.049	0.76	−24.49	−4.26	27.34
Parsopercularis L	–	–	–	0.53 (0.13) vs 0.62 (0.09)	0.049	0.73	48.91	42.83	59.06
Parsopercularis R	41.98 (6.98) vs 46.50 (5.15)	0.049	0.74	0.54 (0.11) vs 0.62 (0.09)	0.049	0.73	−48.83	44.11	57.72
Parsorbitalis R	–	–	–	0.54 (0.12) vs 0.62 (0.09)	0.049	0.72	−43.04	68.62	28.84
Parstriangularis R	40.88 (8.34) vs 45.98 (5.96)	0.049	0.70	0.53 (0.12) vs 0.61 (0.09)	0.049	0.74	−47.74	60.58	45.37
Pericalcarine L	–	–	–	0.54 (0.12) vs 0.62 (0.09)	0.049	0.73	9.33	−55.37	53.56
Pericalcarine R	40.86 (6.50) vs 45.68 (5.47)	0.049	0.80	0.53 (0.11) vs 0.62 (0.09)	0.049	0.80	−12.96	−53.46	54.79
Postcentral L	–	–	–	0.53 (0.12) vs 0.61 (0.10)	0.049	0.78	45.01	4.86	91.16
Postcentral R	41.59 (8.12) vs 46.86 (6.27)	0.049	0.73	0.55 (0.12) vs 0.63 (0.10)	0.049	0.73	−44.52	8.16	91.73
Posterior cingulate L	43.09 (6.95) vs 47.75 (5.70)	0.049	0.73	0.55 (0.11) vs 0.63 (0.10)	0.049	0.72	4.48	12.50	83.85
Posterior cingulate R	43.10 (6.95) vs 47.73 (5.77)	0.049	0.72	0.55 (0.12) vs 0.63 (0.10)	0.049	0.72	−5.75	11.02	84.87
Precentral R	–	–	–	0.53 (0.12) vs 0.61 (0.11)	0.049	0.70	−40.80	21.01	90.91
Rostral anterior cingulate L	–	–	–	0.53 (0.12) vs 0.61 (0.12)	0.049	0.67	4.80	65.47	44.84
Rostral anterior cingulate R	–	–	–	0.53 (0.12) vs 0.61 (0.12)	0.049	0.67	−3.95	64.33	46.63

L, left; R, right.

p value was adjusted by FDR correction.

## Discussion

The present study investigated the effects of 3-week rTMS treatment on the cortical source network in patients with unipolar depression. Significant improvement in symptom severity of depression and anxiety was found after treatment in both active and sham groups. However, increased strength, clustering coefficient, and efficiency in the delta, theta, low-beta, and mid-beta frequency in the global level of the cortical source network were found only in the active group after rTMS treatment. Increased strength and clustering coefficient in mid-beta frequency in nodal levels were also observed after rTMS treatment in the active group.

The improvement in symptom severity of depression and anxiety is an essential clinical indication in the present study. The findings on the treatment effect in the active group could be reasonable; however, it should be clarified whether the significant effects in the active group were due to the actual effects of rTMS treatment. If our study includes the placebo effect, the authentic neurophysiological changes induced by rTMS treatment could be observed in the active group. The small differences of clinical improvement between active and sham group could be explained for psychological expectancy effects to get a treatment (27, 28). All participants might have expected improvements of symptoms from their participation in the present study. Furthermore, a placebo effect could be induced due to daily visits to the hospital during the treatment period in the sham group.

In the global cortical source network, higher values of strength, clustering coefficient, and efficiency in the delta, theta, low beta, and mid beta were found in participants in the active group. Global efficiency in delta frequency could play a modulation role in suppression and amplification of neuronal population, which are implicated in the stabilization of neuronal network during the wakeful state (29, 30), and increased efficiency indicates increased concurrently connected information in the global network. In the global theta network, increased strength, clustering coefficient, and efficiency indicate enhanced dynamics of consciousness, body, and mind, which have a reciprocal relationship with the cooperation system of homeostasis (31). Depressive patients show a reduction in the beta band, which could be recovered after TMS treatment (32). Motor behavior control and executive function are also modulated by beta frequency network changes, which could be induced by functional improvement (32–35).

In the nodal network of the mid-beta band, strength, and clustering coefficient increased after TMS treatment. Previous studies have demonstrated that resting state network could be affected by TMS treatment (36, 37). The default mode network (DMN) involves PCC and parahippocampal gyrus (38), and strength and clustering coefficient of these regions increased after rTMS treatment in the present study. Increased clustering coefficient in the beta band after TMS has been also found in the cortical sensor level (39), but no studies have investigated the effect of rTMS treatment on the cortical source network in patients with unipolar depression. When both the node strength and the sum of weights of connected links increase, the nodal strength is associated with strong assemblies in the network (40); therefore, how neural network changes are related to the underlying treatment effect should be considered. The human brain network has three major functions, i.e., the executive control network (ECN), the salience network (SN), and the DMN (41, 42). In the present study, strength and clustering coefficient in the orbitofrontal region increased after TMS treatment. The frontal cortex, which is the brain area allocating the ECN, is associated with mood regulation in depressive patients (43). Inhibitory function in the orbitofrontal region is implicated in the maintenance of neuronal balance (44). Furthermore, ECN and DMN circuits have a cognitive engagement with episodic memory performance in patients with depression (45). The large parts of insula and ACC have allocated the crucial region of SN, which modulates the shift of a phase between ECN and DMN (46, 47). Therefore, the present study postulates that alterations in nodal network are caused by rTMS treatment and that the function of neural network could be improved in several core regions such as DMN, ECN, and SN.

## Conclusions

In summary, our findings indicate that symptomatic improvement induced by rTMS is accompanied by the altered cortical source networks. The small differences in symptomatic changes between both groups prove that real effects of rTMS treatment on brain networks are an obvious biological evidence in patients with unipolar depression. However, the present study has several limitations. The sample size was small. In addition, the effects of rTMS need to be compared between unipolar depression and other psychiatric disorders.

## Data Availability

All datasets generated for this study are included in the **Supplementary files**.

## Ethics Statement

The studies involving human participants were reviewed and approved by The Institutional Review Board of Seoul St. Mary’s Hospital, College of Medicine, The Catholic University of Korea approved the study protocol (approval number: KC14DDSE0479). The patients/participants provided their written informed consent to participate in this study.

## Author Contributions

K-IJ and J-HC contributed to the design of the research. K-IJ, J-HC and SL contributed to data collection. K-IJ, MS, and J-HC analyzed the data. K-IJ and H-JH interpreted the results and drafted, critically revised, and approved the version to be published.

## Funding

The study was supported by a grant of the Korea Health Technology R&D Project through the Korea Health Industry Development Institute (KHIDI), funded by the Ministry of Health & welfare, Republic of Korea (grant number : HI17C2272 and HL19C0007).

## Conflict of Interest Statement

The authors declare that the research was conducted in the absence of any commercial or financial relationships that could be construed as a potential conflict of interest.
